# Characteristics associated with differences in 24-hour device-measured and self-reported sleep, sedentary behaviour and physical activity in a sample of Australian primary school children

**DOI:** 10.1186/s44167-023-00023-7

**Published:** 2023-07-03

**Authors:** Joshua Gauci, Timothy Olds, Carol Maher, Amanda Watson, François Fraysse, Mason Munzberg, Isaac Hoepfl, Dorothea Dumuid

**Affiliations:** 1 Alliance for Research in Exercise, Nutrition and Activity (ARENA), Allied Health & Human Performance, University of South Australia, City East Campus, Frome Rd, Adelaide, South Australia GPO Box 2471, Australia; 2 Centre for Adolescent Health, Murdoch Children’s Research Institute, Parkville, VIC Australia

**Keywords:** Accelerometry, 24-hour recall, Time use, Sleep, Physical activity, Sedentary

## Abstract

**Background:**

How much time children spend sleeping, being sedentary and participating in physical activity affects their health and well-being. To provide accurate guidelines for children’s time use, it is important to understand the differences between device-measured and self-reported use-of-time measures, and what may influence these differences. Among Australian primary school-aged children, this study aimed to describe the differences between device-measured and self-reported sleep, sedentary behaviour, light-intensity physical activity (LPA), and moderate-vigorous-intensity physical activity (MVPA), and to explore how sociodemographic and personal characteristics were associated with these differences.

**Methods:**

Participants (n = 120, 67% female, age 9–11 years) were drawn from the Life on Holidays cohort study. Device measured use of time was from 7-day accelerometry worn over five timepoints in a 2-year period, and self-reported use of time was from 2-day Multimedia Activity Recall for Children and Adults (MARCA), conducted at the same timepoints. For each participant and measurement method, average daily time spent in sleep, sedentary time, LPA and MVPA was derived for any overlapping days (that had both types of measurement) across the study period. Participant characteristics were either obtained from baseline parental survey (age, sex, parental education, puberty) or derived from the average of direct measurements across the study timepoints (aerobic fitness from shuttle run, body mass index from anthropometric measurements, academic performance from national standardised tests). Differences between device-measured and self-reported use of time were described using Bland-Altmann plots. Compositional outcome linear-regression models were used to determine which participant characteristics were associated with differences by use-of-time measurement type.

**Results:**

Relative to device-measured, self-reported daily LPA was underestimated by 83 min (35% difference), whilst sleep (+ 37 min; 6% difference), MVPA (+ 34 min; 33% difference) and sedentary time (+ 12 min; 3% difference) were overestimated. Characteristics underpinning the differences between measurement types were sex (χ^2^ = 11.9, p = 0.008), parental education (χ^2^ = 23.0, p = 0.001), aerobic fitness (χ^2^ = 10.7, p = 0.01) and academic performance (χ^2^ = 15.9, p = 0.001).

**Conclusions:**

Among primary school-aged children, device-measured and self-reported use-of-time measurements should not be used interchangeably as there are systematic biases and differences relative to socio-demographic characteristics.

**Supplementary Information:**

The online version contains supplementary material available at 10.1186/s44167-023-00023-7.

## Background

How children use their time (sleep, sedentary behaviour and physical activity) affects their health and wellbeing, including body composition, blood pressure, anxiety and depression, academic performance and behaviour [1–3]. Accordingly, governing bodies and health authorities around the world have released public health guidelines and recommendations for how long children should spend being sedentary, sleeping and participating in physical activity each day [4–7]. To better understand the relationships between use of time and health, and to assess and track children’s compliance with the guidelines, epidemiological researchers have developed rigorous methods for measuring how children use their time over a complete day.

There are two broad ways in which 24-hour use of time is typically measured in epidemiological studies: by using a device, such as an accelerometer, or by self-report. Accelerometers are body-worn devices which measure the body’s movement. Typically, cut-points are used to assist in interpreting the raw acceleration data, by grouping it into intensity bands (sedentary time, light-intensity physical activity [LPA] and moderate-vigorous-intensity physical activity [MVPA]) by conversion to different ranges of metabolic equivalent (MET) values [8, 9]. From this, average daily time spent being sedentary, in LPA and MVPA can be determined [8]. Algorithms can be applied to separate sleep time from sedentary time [10]. The other way of measuring use of time is self-report. A recent systematic review found 37 questionnaires for physical activity, sedentary time and sleep, but none had considered a 24-h approach [11]. Twenty-four hour time-use surveys and recalls do consider the 24-hour day, including a widely used and well-validated instrument, the *Multimedia Activity Recall for Children and Adults* (MARCA) [12]. The MARCA is a computerised recall program administered by an interviewer, where participants recall every activity they did over a 24-hour period. Each activity has an estimated energy expenditure, and from this, time spent in sleep, sedentary time, LPA and MVPA can be calculated [13].

However, device-measured and self-reported estimates of sleep, sedentary time and physical activity rarely align closely [14, 15]. There is little research on how children’s device-measured sleep compares to their self-reported sleep, however one study found that children tended to underestimate their total sleep time by around one hour per night [16]. Studies have not compared children’s device-measured and self-reported sedentary time, however a systematic review and meta-analysis by Prince et al. [17] investigated the difference between device-measured and self-reported sedentary time in adults. They found that on average, self-reported sedentary time was around 1.74 h per day less than device-measured sedentary time. A number of studies have reported differences in children’s physical activity measured by device and self-report methods. In particular, MVPA tends to be higher when estimated by self-report than when estimated by devices [15, 18]. Notably, none of the previous exploratory or validation studies have used a 24-hour approach that includes all daily use-of-time behaviours in the same model.

There are several possible reasons why children’s device-measured and self-reported use of time might differ. First, social desirability bias may cause children to report activity levels to portray themselves positively [19, 20] Second, children may have difficulty remembering durations of activities. According to psychologist Jean Piaget’s work, the concept of time develops from a young age, however, to estimate durations correctly, they must be forced to pay attention to time [21]. This can be difficult during activities such as reading or video gaming, where attention is on the task [22]. The skill of accurately estimating time is still developing during the “concrete operational stage of cognitive development”, between 7 and 11 years-of-age [21]. Third, recall bias (i.e., the failure of memory) may lead to children defaulting to “typical” rather than actual days [23]. Fourth, self-report measures may be subject to epoch effects, where children tend to remember blocks of time. For example, someone may recall a game of tennis lasting an hour (60 min reported as MVPA), but they were only active for half an hour, or a movie going for 90 min (reported all as sedentary time), but they were actually active during advertisement breaks [24]. Epoch effects could also cause differences in sleep, where self-reported sleep may be remembered as being between bedtime and morning wakening, without considering night awakenings which accelerometers can track. Fifth, self-report measures may suffer cutpoint bias, where the combination of inter- and intra-variability in energy expenditure estimates and a high proportion of activities at or just above the traditional 3 MET cutpoint results in systematic over-estimates of MVPA [18]. Lastly, processing accelerometry data requires a series of relatively arbitrary choices around body placement, sampling frequency, filtering, computation of acceleration magnitude, cutpoint values, and non-wear and sleep definition and detection [24, 25]. This means that, depending on the choices made, a very wide range of estimates of use of time can be derived from the same person over the same period. Understanding the factors that influence the divergence of device-measured and self-reported use of time may help to explain inconsistencies across study findings and provide indications on how the two types of measures could be harmonised.

Only a few studies have explored what leads to differences between device-measured and self-reported use-of-time estimates among children, and their findings suggest sociodemographic factors play a role. A study by Slootmaker et al. [26] found that boys had larger differences between device-measured and self-reported physical activity than girls. Age may also have an influence, with children aged 7–11 considered to still be developing their concept of time, which could influence their ability to validly complete use-of-time recalls [21]. The difference between device-measured and self-reported use of time estimates may be smaller in children with a higher academic ability or parental education level, as they could have a better understanding of time [27] and may be less susceptible to recall bias. There have been mixed findings on whether body mass index (BMI) impacts use of time reporting [28], and along the same lines, a child’s fitness level may play a role [29]. It remains mostly unknown how sociodemographic and personal factors influence the divergence between device-measured and self-reported use of time.

Time spent in sleep, sedentary time, LPA and MVPA can differ significantly depending on how use of time is measured. Given the importance of use of time to child health and well-being outcomes, there is a need for a better understanding of the differences and the potential characteristics driving these differences in measurement to be able to provide robust time-use recommendations to families.

The aim of this study is to describe the similarities and differences between device-measured and self-reported 24-hour use of time in primary school-aged children, and to determine whether differences in measurement are associated with socio-demographic and personal characteristics.

## Methods

### Research design

This was an observational, cross-sectional study. Data were drawn from a 2-year, longitudinal cohort study *Life on Holidays: fitness lost, fatness regained?* [30]. Participant characteristics were obtained from the first timepoint of the two study waves, except where they were unavailable, in which case the second timepoint was used. Matched accelerometry and MARCA days were drawn from any timepoint. Reporting of study findings followed the STROBE framework. The STROBE checklist is included in Supplementary File 1.

Ethical approval was obtained from the University of South Australia Human Research Ethics Committee, Adelaide, Australia (200980), the South Australian Department of Education and Child Development (2008-0055) and Catholic Education South Australia (201820).

### Sample selection

Participants were in grade four at the time of recruitment, attending a mainstream Government, Catholic or Independent primary school in the greater Adelaide (South Australia) area. All eligible schools (n = 334) were grouped into socioeconomic position tertiles based on their Index of Community Socio-Educational Advantage (ICSEA) score [31]. Schools were randomly selected from each ICSEA tertile, with school principals needing to agree to be a part of the study for the school to be included. All grade four children were invited to participate. Written consent forms were signed and returned by a parent/guardian of each participant, and verbal assent to proceed with a test or measurement was obtained from participants. Any child, or parent/guardian on behalf of the child, could withdraw from the study at any time without penalty. Recruitment of schools and students continued until at least 100 students were recruited from each of the three socioeconomic tertiles. Data were collected from Term one, 2019 (beginning 29th January) onwards for wave one schools, and Term one, 2020 (beginning 28th January) onwards for wave two schools.

### Procedures and measurement tools

Data were collected during school visits and phone interviews with parents/guardians.

#### Use of time

Device-measured use of time (sleep, sedentary time, LPA and MVPA) was obtained from GENEActiv accelerometers (Activinsights, Cambridgeshire, UK). The GENEActiv has strong intra-instrument and inter-instrument reliability (CV_intra_=1.4% and CV_inter_=2.1%), very good test-retest reliability (ICC = 0.67–0.87) [32], and excellent convergent validity (r = 0.98) [33].

Devices were configured to record triaxial acceleration continuously at 50 Hz, starting at midnight on the day the device was handed to the participant. Participants were instructed to wear the device on their non-dominant wrist for seven consecutive days, and to only take the device off for prolonged water immersion. Participants were also asked to complete a paper log each day, indicating their get-up time, bed time, and any time they took off the device, with an option to provide a reason for the removal.

After the devices were collected by the research team, raw data was downloaded using the Geneactiv PC software (Activinsights, UK). The acceleration magnitude (SVM) [33] was computed as the absolute value of the Euclidean norm of the acceleration signal, minus 1 g (1 g = 9.81 m/s^2^):

$$SVM= \left|\sqrt{{a}_{x}^{2}+{a}_{y}^{2}+{a}_{z}^{2}}-g\right|$$

The SVM was then summed over 1-minute epochs for analysis. Further processing was performed using bespoke software (“Cobra”) developed in-house in Matlab, previously used in other studies [34, 35].

First, sleep periods were identified automatically from the participants’ self-report logs. Bed and get-up times were corrected by visual inspection if needed, or in case of incomplete logs.

Second, non wear periods were identified in the same manner. In case the reason provided for device removal was “sports”, the non-wear period was replaced by a mix of 40% MVPA, 40% LPA and 20% sedentary time, according to the findings of Ridley et al. [36] regarding children’s activity patterns during organised sports. In Australia, children are usually asked to take off jewellery items and/or watches for team sports; as such, we decided that replacing these non-wear periods with a representative mix of PA levels would give a better estimate of children’s activity.

Third, all waking wear epochs (i.e. not previously classified as sleep or non-wear as per the process above) were classified as either sedentary, light physical activity (LPA) or moderate-to-vigorous physical activity (MVPA) according to cut-points developed by Philips et al. [8] for children. In the Philips study, sedentary was defined as energy consumption < 1.5 METs, LPA as 1.5–2.99 METs and MVPA as ≥ 3 METs.

A single day was considered valid if it had at most 6 h of non-wear, and at least 10 h of waking wear time. A participant was considered valid if they had at least 4 valid days including at least one weekend day.

Self-reported use of time was measured using the MARCA [12], a recall instrument administered by a computer-assisted in-person or telephone interview. A trained interviewer asked the participant to recall every activity they did over the last two days, with five minutes being the minimum time interval. The MARCA program contains over 500 activities to choose from, with estimated energy expenditures for each [13], so that average daily time spent in sleep, sedentary time, LPA and MVPA could be estimated. The MARCA has been validated against accelerometry, pedometry and doubly-labeled water (validity: r = 0.4–0.7, test-retest reliability: ICC = 0.88–0.94) [12, 37, 38].

#### Participant characteristics

Age, sex, parental education and pubertal status: Data were collected through a questionnaire completed by the parent/guardian at baseline. The highest household parental education level, classified as either low (year 12 or less), mid (vocational training) or high (Bachelor’s degree or postgraduate qualification), was used as a proxy for socioeconomic position (SEP). Pubertal status was classified as either pre-pubertal, early puberty, mid-pubertal, late puberty or post-pubertal using the Pubertal Development Scale [39]. The scale has been validated against Tanner Staging (r = 0.54–0.79) and has good test-retest reliability (ICC = 0.81–0.92) [40].

For academic performance, aerobic fitness and body mass index, we used the average of available data across the study timepoints to match up as much as possible with the time-use measurements.

Academic performance: Progressive Achievement Tests (PAT) provide objective measures of academic achievement [41]. All government schools and most independent and Catholic schools sit two tests, PAT reading and PAT mathematics, in October each year as part of a routine assessment. PAT test data from Government schools were provided by the Department for Education, and Catholic and independent schools provided data for their students. Scaled scores for PAT reading and PAT mathematics were averaged to determine overall academic performance. A higher score indicated better academic performance. Progressive Achievement Tests have been validated against school grades in boys (r = 0.53–0.87) and girls (r = 0.52–0.73) [42].

Aerobic fitness (VO_2_max): VO_2_max is an indicator of aerobic fitness and was assessed using the 20-m shuttle run test [43]. The test involved participants running between cones set up 20 m apart, on each beep. When the participant failed to reach the cones before the beep twice in a row, they ceased the test and their level reached was recorded. The child’s final score was used to estimate their VO_2_max using the equation outlined by Nevill et al. [44]. As a predictor of VO_2_max, the 20-m shuttle run test has been validated against a treadmill VO_2_max test (r = 0.69) and has good test-retest reliability (ICC = 0.78–0.93) [45, 46].

Body mass index (BMI): Each child’s height was measured to the nearest 0.1 cm using a Seca 213 stadiometer (Seca, Hamburg, Germany), and weight measured to the nearest 0.1 kg, using the InBody 270 Bioelectrical Impedance Analyser scales (InBody USA, California, USA) without shoes and in light clothing. Measures were taken twice, and a third measure taken if there was more than 0.5 cm or 0.5 kg difference between the first two measurements. The mean of the two measurements, or median of the three if three were done, was used in the analysis. From these measurements, BMI was calculated using the formula: BMI = (weight in kg)/(height in m)^2^. A z-score for each child’s BMI was calculated against World Health Organization standards [47]. The methods used to measure height and weight both have excellent intra- and inter-rater reliability (ICC > 0.96) [48].

### Statistical analysis

MARCA recalls that captured use-of-time data while the participants were wearing the accelerometer (and had valid data for that day) were extracted only if the dates could be matched. Of a total of 7819 valid accelerometer days and 2307 MARCA profiles, there were 312 matched days across 133 participants. Two of these were excluded due to very low MARCA sleep durations (< 300 min/d). Use of time estimated from the MARCA and accelerometery on matched days were averaged, so that there was one “average” MARCA day, and one “average” accelerometry day per participant. We chose to use averages rather than time-varying variables to create the most parsimonious model to answer our research question. Accelerometer non-wear time (24 min/d, SD = 45) was accounted for by linearly adjusting all the use-of-time variables to collectively sum to 1440 min (24 h).

Variables were described using means and standard deviations for continuous and normally distributed data. Categorical variables were described using counts and percentages.

#### Single movement behaviour analyses

Similarity between measurement methods for each movement behaviour was assessed by calculating intra-class correlation (ICC) estimates and their 95% confidence intervals, using the R Psych package [49]. This was based on a “single-rater” unit (self-reported use of time was compared to device-measured), absolute-agreement, two-way random effects model. ICC estimates were determined for each behaviour (sleep, sedentary time, LPA and MVPA) and classified using criteria given by Koo and Li [50]. Differences between measurements were assessed using Bland-Altman plots.

#### Compositional (multi-variable) analyses

The single movement behaviour analyses were exploratory as they did not consider that the differences in measurement of one variable will necessarily be co-dependent on the differences in measurement of at least one of the other variables. For example, if the self-reported measurement for sleep was higher than the device measurement, then the self-reported measurement for one or more other variables must be lower than the respective device measurement. Similarly, if the estimates for one movement behaviour are quite similar, it is more likely that the others will be similar. The co-dependency between the measures occurs because use-of-time data are compositional; they are made up of mutually exclusive and exhaustive components (sleep, sedentary time, LPA and MVPA) which sum to a constant whole (24 h). A specific statistical approach called compositional data analysis (CoDA) is required for compositional data [51].

Using a CoDA framework, the activity data were considered as 24-hour use-of-time compositions comprising time spent in sleep, sedentary time, LPA and MVPA [52]. The complete device-measured and self-reported use-of-time compositions were used in one model. The use-of-time compositions were expressed as a set of isometric log-ratios, which enabled the co-dependency between the activities to be accounted for [53]. A multi-level linear regression model was used, where each participant had two dependent compositions (their average device-measured and self-reported use-of-time compositions, expressed as a set of isometric log ratios) [54]. A stacked model format was required to run the multi-variate model (isometric log ratios stacked within participant ID), with random slopes at the log-ratio level. The model tested for an interaction effect between potential correlates of differences in measurement (sociodemographic and personal factors) and a categorical variable representing the type of composition (device-measured or self-reported). A nested random intercept accounted for the nesting of observations within participants, schools, and study waves. A random intercept model was used to account for the fact that the self-reported and device-measured estimates from one particular child (and school, and study wave) are more likely to be similar than those from two different children (and schools, and study waves). A significant interaction effect (p < 0.05) identified which factors were correlated with divergence in measurement of the overall compositions. To explore which activities were driving the divergence (and how), the multi-level models were used to estimate (adjusted the nested study design) device-measured and self-reported compositions for the different levels of the correlate in question (e.g., boys vs. girls, or high vs. low academic performance). The model-based estimates were plotted to aid interpretation.

## Results

**Fig. 1:**
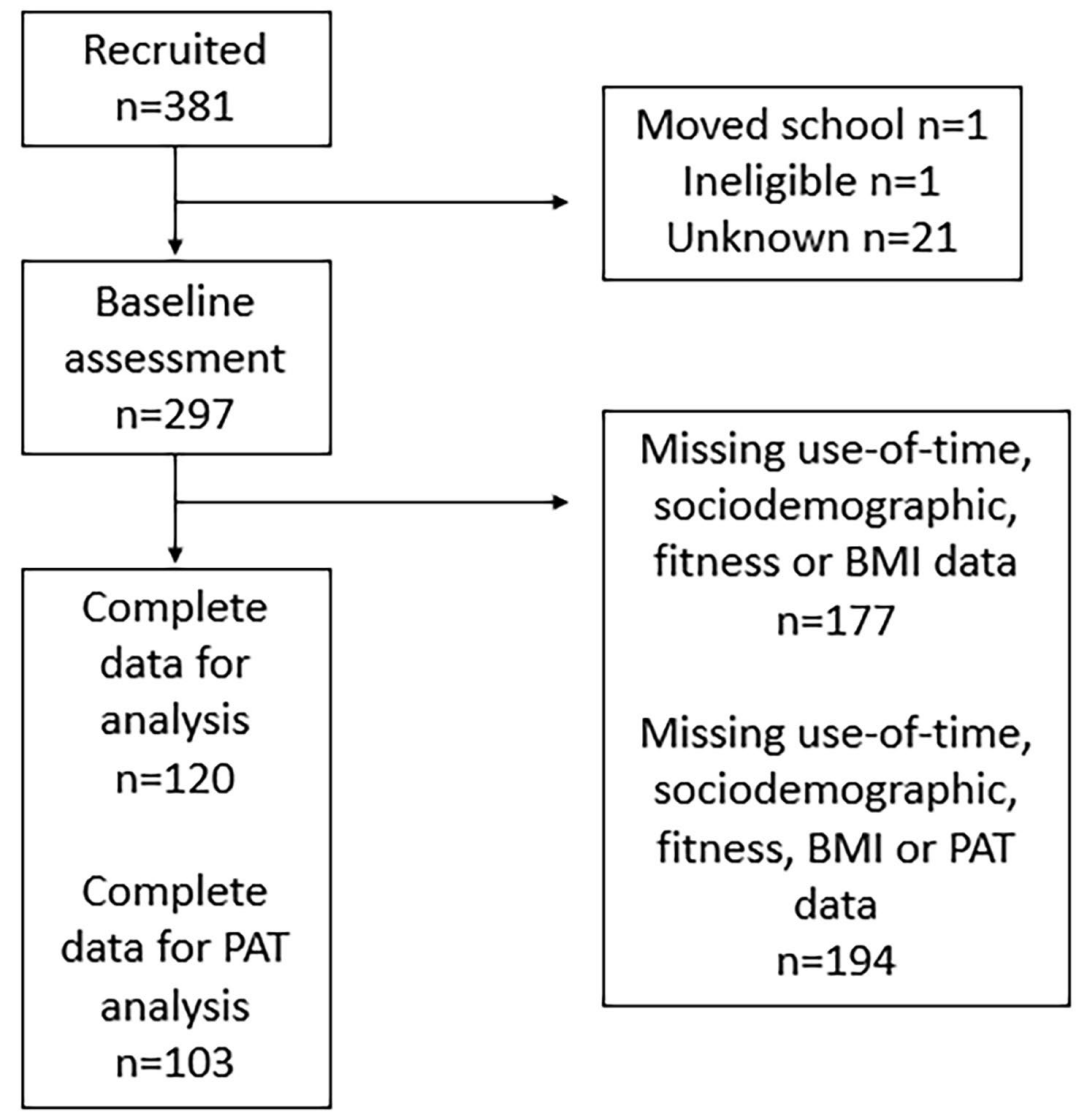
Participant Flow Abbreviations: PAT: progressive achievement test, BMI: body mass index

A total of n = 120 participants had at least one matched day of device measured and self-reported use of time, as well as complete demographic and personal characteristic data. Participant characteristics and descriptive summaries of use-of-time variables on matched days are presented in Table 1. Participant flow is shown in Fig. 1. Compared to excluded participants, the included sample had more females (p = 0.01), older age (p = 0.002), higher parental education (p = 0.002), lower zBMI (p = 0.004), higher academic performance (p = 0.001) and higher accelerometer-measured sleep (p = 0.005). Descriptive summaries of the samples can be found in Supplementary Table 1.

**Table 1:** Participant characteristics

		All	Females	Males
Baseline Characteristics				
	n (%)	120 (100)	82 (68)	38 (32)
	Age (mean (SD))	10.0 (0.3)	10.0 (0.3)	10.1 (0.3)
Puberty Stage (n (%))				
	Pre-pubertal	78 (65)	43 (52)	35 (92)
	Early pubertal	24 (20)	21 (26)	3 (8)
	Mid-pubertal	18 (15)	18 (22)	0 (0)
Parental Education (n (%))				
	Low	8 (7)	7 (9)	1 (3)
	Mid	43 (36)	28 (34)	15 (40)
	High	69 (58)	47 (57)	22 (58)
Fatness/Fitness^a^ (mean (SD))				
	zBMI	0.17 (1.20)	0.10 (1.21)	0.33 (1.18)
	VO_2_max (mL/kg/min)	40.1 (6.6)	37.4 (4.1)	45.9 (7.1)
Academic Performance (mean (SD))				
	PAT scaled score	126 (9)^n=103^	127 (9)^n=69^	123 (9)^n=34^
Device-Measured (Accelerometry)^b^ (arithmetic mean (SD)) (min/day)				
	Sleep	588 (52)	585 (56)	595 (41)
	Sedentary	488 (98)	502 (92)	458 (105)
	LPA	279 (70)	278 (70)	280 (70)
	MVPA	85 (52)	75 (44)	106 (62)
Self-Reported (MARCA)^b^ (arithmetic mean (SD)) (min/day)				
	Sleep	625 (62)	619 (65)	637 (53)
	Sedentary	501 (105)	500 (107)	503 (102)
	LPA	196 (74)	202 (79)	182 (62)
	MVPA	119 (64)	119 (67)	118 (58)
MARCA minus Accelerometry (mean (SD)) (min/day)				
	Difference in sleep	37 (59)	34 (65)	41 (44)
	Difference in sedentary	12 (109)	-3 (106)	45 (109)
	Difference in LPA	-83 (91)	-76 (91)	-98 (89)
	Difference in MVPA	34 (68)	44 (71)	12 (58)

NB Accelerometer use-of-time data were normalised to sum to 1440 min

^a^ Measures were from first available time point

^b^ Average of all available matched (MARCA and accelerometry) days from any time point

Abbreviations: zBMI: body mass index z-score; VO2max: maximal oxygen consumption; LPA: light physical activity; MVPA: moderate-to-vigorous physical activity; MARCA: Multimedia Activity Recall for Children and Adults; PAT: progressive achievement test

### Single movement behaviour analyses

There was poor-moderate agreement between device and self-reported measurements for all use-of-time behaviours (all ICC < 0.5; Table 2).

**Table 2:** Intraclass correlation (ICC) and 95% confidence intervals (CI) for device and self-reported measurements

Behaviour	ICC	Lower CI	Upper CI	F-test (true value = 0)	p
Sleep	0.38	0.13	0.57	2.73	< 0.001
Sedentary	0.43	0.27	0.56	2.50	< 0.001
LPA	0.12	-0.05	0.29	1.51	0.01
MVPA	0.27	0.08	0.43	1.89	< 0.001

Abbreviations: LPA: light physical activity; MVPA: moderate-vigorous physical activity

**Fig. 2:**
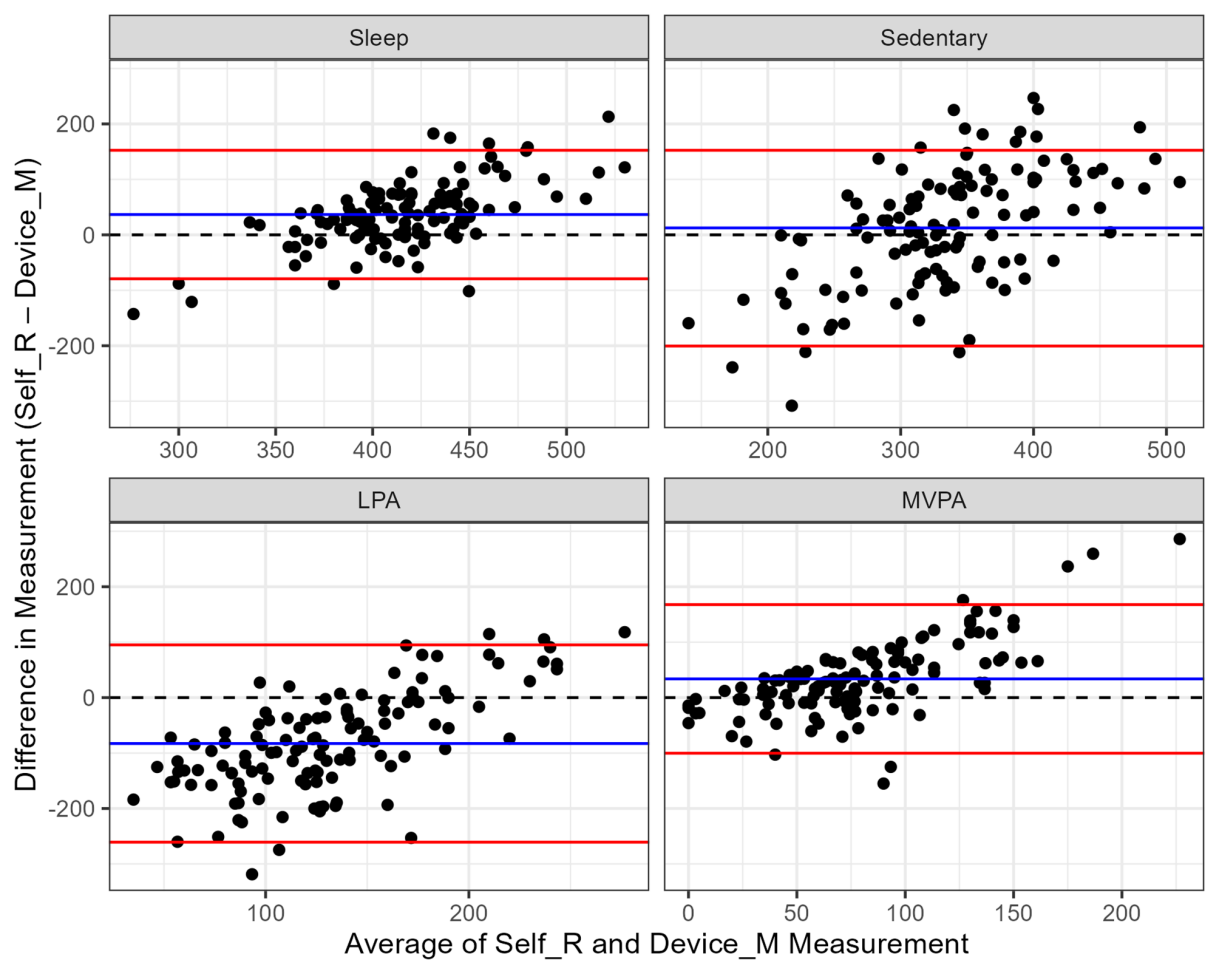
Bland-Altman Plots for differences in measurement of each behaviour NB Average measurement (x-axis) refers to the average of device measured (accelerometry) and self-reported (MARCA) use of time Blue line: average/bias; red lines: 95% confidence interval Abbreviations: LPA: light physical activity; MVPA: moderate-to-vigorous physical activity; Self-R: self-reported (MARCA); Device-M: device-measured (accelerometer); MARCA: Multimedia Activity Recall for Children and Adults

Bland-Altman plots (Fig. 2) revealed the greatest differences in measurement for LPA, with a bias of -83 min/day, i.e. the self-reported measurements were on average 83 [95% limits of agreement: -79; 153] min/day lower than the device-measured measurements. The difference in time was regained from the remaining behaviours, with self-reported use of time being higher than device-measured for sleep (+ 37 [-260; 95] min/day), MVPA (+ 34 min/day [-100; 168]) and sedentary time (+ 12 [-200; 225] min/day). Limits of agreement were most narrow for sleep and widest for sedentary time. The upward trend of the points in the plots indicates that as the time spent in each behaviour increased, the difference between device-measured and self-reported time also increased for each behaviour. Differences were largest for MVPA according to Mean Absolute Percentage Error (MAPE) and considering the self-reported value as the “forecast” value, and the device-measured value as the “actual” value. The MAPE for MVPA was 108%, compared to 36% for LPA, 19% for sedentary time and 9% for sleep.

### Compositional use-of-time analyses

In relation to use-of-time composition, statistically significant interactions between the use-of-time measurement method and participant characteristics were found for sex (p = 0.008), parental education (p = 0.001), VO_2_max (p = 0.01) and academic performance (0.001), as shown by the omnibus analysis of variance (χ^2^) tests presented in Table 3.

**Table 3:** Multi-level regression model interaction terms and estimates of use-of-time compositions for both measurement types (n = 120)

Factor	Interaction: Type-by-characteristic		Level	Model-based estimates of device-measured use of time				Model-based estimates of self-reported use of time			
	χ^2^	p		Sleep	Sedentary time	LPA	MVPA	Sleep	Sedentary time	LPA	MVPA
**Sex**	**11.9**	**0.008**	Male	616	464	277	83	659	512	176	93
			Female	600	509	272	60	646	511	192	91
Age (y)	0.6	0.89	9.5	614	493	268	66	659	519	171	91
			10	604	492	277	67	650	510	188	92
			10.5	595	491	287	68	640	501	206	94
Puberty	11.5	0.08	Pre	606	485	278	71	649	512	186	93
			Early	597	504	272	67	650	523	183	84
			Mid	602	513	275	51	646	489	205	100
**Parental Education**	**23.0**	**0.001**	Low	602	523	268	47	678	533	174	55
			Mid	605	481	291	64	652	510	170	108
			High	601	493	274	72	641	503	206	89
zBMI	0.2	0.97	-1SD	592	504	277	67	643	514	190	93
			mean	604	493	276	67	649	511	188	92
			+ 1SD	615	482	276	67	655	507	187	92
**VO**_**2**_ **max**	**10.7**	**0.01**	-1SD	594	523	270	52	651	522	185	83
			mean	603	492	277	69	648	509	188	95
			+ 1SD	608	460	282	90	644	496	192	108
**PAT Score (n = 103)**	**15.9**	**0.001**	-1SD	605	488	278	69	653	515	193	79
			mean	602	502	271	65	647	515	188	90
			+ 1SD	598	517	263	62	641	514	183	103

NB: All models are adjusted for repeated measurements within participants, schools, and data collection waves using random intercepts. Statistically significant (p < 0.05) characteristics are shown in bold

Abbreviations: χ^2^: chi-squared test; zBMI: body mass index z-score; VO2max: maximal oxygen consumption; PAT: progressive achievement test (academic performance); LPA: light physical activity; MVPA: moderate-to-vigorous physical activity

**Fig. 3:**
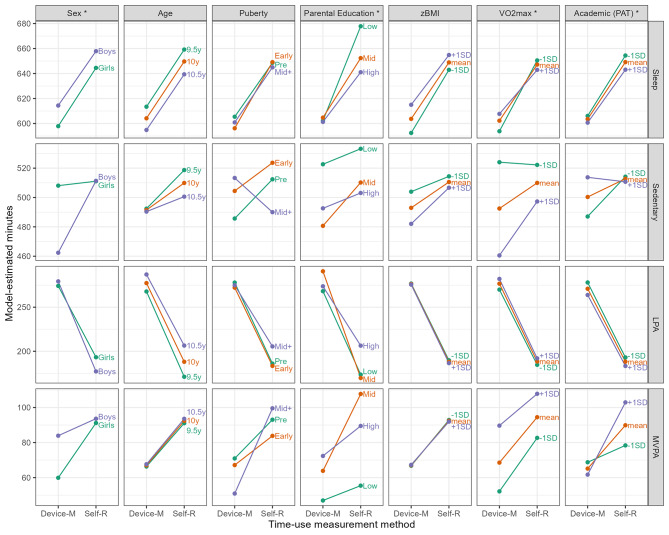
Model estimated use-of-time compositions for device and self-reported measurement methods across child characteristics (n = 120) NB. All models are adjusted for repeated measurements within participants, schools, and data collection waves using random intercepts. Random slopes for ILR numbers are included due to the long model format. Significant interaction effects are indicated by an asterisk (*) in the column header. In the academic performance (PAT) model, the sample size is n = 103. Abbreviations: Self-R: self-reported (MARCA); Device-M: device-measured (accelerometer); MARCA: Multimedia Activity Recall for Children and Adults, zBMI: body mass index z-score; VO2max: maximal oxygen consumption; PAT: progressive achievement test; LPA: light physical activity; MVPA: moderate-to-vigorous physical activity

Figure 3 shows the model-estimated use-of-time compositions for both measurement types. If a plotted line in any panel is on an upward incline, this means that the self-reported estimate is higher than device-measured for that characteristic. For example, looking at sleep by sex, both the lines are on an upward incline, meaning there was more self-reported sleep than device-measured for both boys and girls. Variables can be grouped into four categories: converging, diverging, isomorphic or inverting. Looking at self-reported time relative to device-measured, the converging variables’ lines on the graph approach each other, such as sedentary time by sex and by PAT. This means that the difference in minutes between the variables of that characteristic is less for self-reported time than for device-measured time. For diverging variables, the lines become further apart from each other, such as sedentary time by age. This means that the difference in time between the variables of that characteristic is more for self-reported use of time than for device measured. For isomorphic variables, the lines stay relatively parallel, meaning the relationships from device-measured to self-reported use of time are maintained across the characteristic, such as sleep by age and LPA by zBMI. For inverting variables, the lines cross, such as MVPA by PAT, meaning the relationships are reversed. Participants with a lower PAT average score had more device-measured MVPA than those with a higher average score, however participants with a higher PAT self-reported more daily MVPA.

## Discussion

This study found that there were differences between device-measured and self-reported use of time among Australian primary school children. Compared to device-measured use of time, self-report underestimated daily LPA by 83 min, and compensated by overestimating sleep (+ 37 min), MVPA (+ 34 min) and sedentary time (+ 12 min). All these differences appear meaningful and clinically significant, when considering the effect sizes commonly reported in child behaviour interventions. For example, a systematic review of child physical activity intervention studies reported sustained (6-month) increases in MVPA of 1.47 min/day, and under 5 min/day for all subgroup analysis [55]. A systematic review of child sedentary behaviour interventions reported 17.12 min/d reductions in children’s screen time and 18.91 min/d reductions in non-screen sedentary time [56]. The characteristics of the participants which underpinned the differences between measurement types were sex, parental education, aerobic fitness and academic performance.

The finding of differences between device-measured and self-reported use of time among children is consistent with previous studies [15, 26]. Maddison et al. [15] compared MARCA-reported LPA and MVPA to accelerometry-measured LPA and MVPA. Consistent with our study, they reported overestimation of self-reported MVPA, however the magnitude was almost four times that in this study; +124 min/day in boys and + 114 min/day in girls. They also reported differences in LPA, but these were smaller than in this study and differed by sex (boys − 21 min/day; girls + 13 min/day) [15]. A key reason which may explain the differences was that the study by Maddison et al. [15] used hip-worn ActiGraph accelerometers and Freedson’s [57] cut-points to measure use of time, whereas this study used wrist-worn GENEActiv accelerometers with Phillip et al’s cutpoints [8]. Differences between the use-of-time measures was also consistent with other studies done with an adult population [14, 17].

Previous studies by Maddison et al. [15] and Olds et al. [18] found that boys tend to overreport their MVPA compared to device-measures more than girls. In contrast, this study found that girls’ self-reported MVPA was 31 min higher than device-measured, whereas boys’ self-reported MVPA was only 10 min higher. The contrasting findings may be because this study’s sample was younger than that of Olds et al. [18], where the population was aged 16 and over, and that the study by Maddison et al. [15] was undertaken using a different accelerometry protocol (different brand of accelerometer, different wear site, and different cut-points). Another important factor when comparing this study to others would be that the results were obtained using compositional models which include all use-of-time behaviours concurrently and accounts for the fixed 24-hour daily total. To the authors’ knowledge, a compositional 24-hour use-of-time study in children comparing device-measured and self-reported use of time has not been done previously.

There is little research on participant characteristics associated with the differences between device-measured and self-reported use of time. A study by Slootmaker et al. [26] investigated characteristics driving the differences between device-measured and self-reported MVPA, but this was not done compositionally. Consistent with the Slootmaker et al. [26] study, this study found that children with better academic achievement self-reported more MVPA but those with lower academic achievement had more device-measured MVPA. The influence of parental education level, aerobic fitness and other factors have not previously been examined so it was not possible to compare the findings to previous studies.

A reason why large absolute differences in LPA between measurement types were found could be due to epoch effects. The application of MET-equivalents to MARCA activities does not account for some of the time during an activity spent in other energy expenditure bands. For example, the MARCA classifies sport as MVPA. However, using accelerometers, Leek et al. [58] found that during sports practice (soccer), 28% was spent sedentary, 19% in LPA and only 53% in MVPA [58]. Whilst MVPA is dominant, it only accounts for just over half of the duration of the activity. Light PA can also occur within blocks of sedentary time, such as standing up and walking to the toilet, getting food or drinks, and even standing and talking. Accelerometers can recognise when these disruptions to sedentary time occur and attribute LPA, however people may only recall two hours of watching television and forget the time they were standing up and moving. These two reasons may contribute to why device-measured LPA was much higher than self-reported. Epoch effects could also be a reason that self-reported sleep values were higher for all characteristics, as participants may not have accounted for, or remembered, time awake in bed during the recall interviews.

Accelerometer classification of activities is very dependent on decisions made by the research team, such as where the energy expenditure cutpoints are placed. There may be misclassification where sedentary time or MVPA is classified as LPA. Also, the relatively long epoch length of 1-minute used in this study may misclassify some sporadic MVPA as LPA, resulting in lower device-measured MVPA [24].

### Strengths and limitations

A key strength of this study is its use of compositional data analysis to account for the co-dependency of the 24-hour use-of-time behaviours. The study’s high-fidelity, validated measurement methods and standardised protocols are another strength.

One limitation of this study is that there were relatively few participants with matched days of both measurement types, resulting in a relatively small sample size, particularly in the low parental education level category. Another potential limitation is that non-wear time during accelerometry days was accounted for by linearly adjusting each behaviour so that the total minutes equated to 1440 (24 h). This may have been an inaccurate way of dealing with non-wear time, as for example, it may have all been spent sedentary (e.g. in the bath), or participating in sport (LPA and/or MVPA). We partly addressed this by replacing all non-wear periods identified as sport (from logs) with a pre-determined mix of MVPA, LPA and sedentary time, but the mix of these activities could vary widely depending on the sport being played. However, the average wear time was 1416 (SD = 45) minutes per day, just 24 min less than a full daily 1440 min, meaning the effect on the compositional analyses is expected to be minimal. Finally, the age range of the participants was very narrow (10.1 ± 0.3 years), and these findings may not generalise to other age groups with different cognitive capacities.

### Implications

Our findings suggest that there are some participant characteristics that may influence the difference between children’s device-measured and self-reported use of time. In particular, the role of children’s sex, parental education, aerobic fitness and academic performance should be considered when selecting covariates for models involving use-of-time compositions. Future studies should clearly report which measurement method was used to determine guideline compliance, and technical documents supporting movement behaviour guidelines should specify which measurement methods their recommended durations were derived from.

## Conclusion

In this study, differences were found between device-measured and self-reported use of time among Australian primary school children. Self-report underestimated daily LPA by 83 min, and compensated by overestimating sleep, MVPA and sedentary time, compared to accelerometry. Four characteristics of the participants underpinned the differences; sex, parental education, aerobic fitness and academic performance. The findings suggest that, among primary school-aged children, device-measured and self-reported use-of-time measurements should not be used interchangeably as there are systematic differences, especially for LPA. The bias in sleep and MVPA was lower but could have substantial implications for assessing whether or not children meet movement behaviour guidelines.

## Electronic supplementary material

Below is the link to the electronic supplementary material.

Supplementary Material 1

## Data Availability

The datasets used and/or analysed during the current study are available from the corresponding author on reasonable request.

## Abbreviations

**List of abbreviations**
LPA: Light physical activity
MVPA: Moderate-to-vigorous physical activity
MET: Metabolic equivalent
MARCA: Multimedia Activity Recall for Children and Adults
BMI: Body mass index
ICSEA: Index of Community Socio-Educational Advantage
PAT: Progressive Achievement Tests
VO2max: Maximal oxygen consumption
CoDA: Compositional data analysis
